# Characteristics and real-world medication persistence of people living with HIV treated with DTG/3TC or BIC/FTC/TAF: a hospital claims database study in Japan

**DOI:** 10.3389/fmed.2024.1329922

**Published:** 2024-09-10

**Authors:** Rie Kanamori, Nozomi Aoki, Akio Kanazawa, Mayumi Yuda, Nao Makino, Emi Ohata, Nobuyuki Fukui, Hirotake Mori, Hirohide Yokokawa, Toshio Naito

**Affiliations:** ^1^Department of General Medicine, Juntendo University Faculty of Medicine, Tokyo, Japan; ^2^Center for Promotion of Data Science, Juntendo University Graduate School of Medicine, Tokyo, Japan

**Keywords:** HIV, antiretroviral therapy, single-tablet regimen, treatment pattern, medication persistence, real-world, characteristics, aging society

## Abstract

**Background:**

As the life expectancy of people living with human immunodeficiency virus (HIV) (PLWH) has improved, chronic disease burden and polypharmacy have increased in PLWH. Simplification of the antiretroviral therapy (ART) regimen for PLWH has become crucial. The real-world treatment patterns and medication persistence of the 2-drug single-tablet regimen (STR), dolutegravir/lamivudine (DTG/3TC), compared to bictegravir/emtricitabine/tenofovir alafenamide (BIC/FTC/TAF) prescribed were investigated.

**Methods:**

This retrospective, database study extracted data from a hospital-based medical claims database in Japan. The changes in ART distributions by year during the identification period between January 1, 2018 and December 31, 2021 were observed. Patients with disease record of HIV-1 infection and prescribed DTG/3TC or BIC/FTC/TAF as the first prescription of STR during the identification period were divided into two cohorts; DTG/3TC cohort and BIC/FTC/TAF cohort, respectively. Patient without medication records more than 3 months and no future data more than 6 months were excluded. Patients’ characteristics were compared between the DTG/3TC cohort and the BIC/FTC/TAF cohort by Mantel–Haenszel test to adjust for age. Medication persistence was compared between the two cohorts by evaluating the continuation rates using Kaplan–Meier methods, using the log-rank test to assess the difference between the Kaplan–Meier curves. The median time-to-first prescription was compared between the two cohorts by Kaplan–Meier methods.

**Results:**

Prescriptions of DTG/3TC and BIC/FTC/TAF increased steadily from 2019 to 2021 after the release year of each STR. There was no significant difference in the time-to-first prescription (*p* = 0.3). A total of 959 patients were included, with 120 patients and 839 patients on DTG/3TC and BIC/FTC/TAF, respectively. The proportion of dyslipidemia at baseline was significantly higher in the DTG/3TC cohort than in the BIC/FTC/TAF cohort after adjusting for mean age (*p* = 0.002). There was no significant difference in medication persistence between the two cohorts (*p* = 0.91).

**Conclusion:**

This study showed that DTG/3TC was likely to be selected for elderly patients and those with chronic disease in real-world clinical practice, which seems in accordance with the treatment strategy recommended by guidelines. Comparable medication persistence was observed with both regimens, aligning with findings from other countries. The 2-drug single-tablet regimen DTG/3TC may be an important ART regimen for PLWH with multiple morbidities and polypharmacy in an aging society. Due to the limitations of the database, further research to assess viral loads, emergence of resistance and adverse events will be encouraged.

## 1 Introduction

With the development of more effective antiretroviral therapy (ART) for human immunodeficiency virus (HIV) infection, life expectancy of people living with HIV (PLWH) has recently been prolonged (1–3), and they have been aging as well as people without HIV. Our previous studies demonstrated that the number of chronic comorbidities and concomitant medications in PLWH increases with age in Japan (4, 5). Thus, simplification of the ART regimen, such as single-tablet regimen (STR), for PLWH has become crucial to alleviate concerns of drug–drug interactions, and long-term toxicity with improving tolerance and safety.

The approval of the 2-drug STR (dolutegravir/lamivudine, DTG/3TC), which includes INSTI, has prompted a change in trends of ART regimen use prescribed globally. Our previous studies showed that integrase strand transfer inhibitors (INSTIs) may be the most durable anchor drug for PLWH (6, 7). The efficacy and safety of DTG/3TC have been reported in clinical trials in treatment-naïve (8, 9) and treatment-experienced PLWH (10, 11), which indicates that this regimen is suitable for use as a 2-drug STR. Even though switching to dual therapy DTG/3TC was reported as non-inferior to continuing tenofovir alafenamide (TAF)-based 3-drug regimens for maintaining virological suppression in some clinical trials, concerns about using DTG/3TC as an initial regimen have been reported, including resistance emergence associated with lack of a second non-nucleoside reverse transcriptase inhibitors (NRTIs) (12). The risk of discontinuation due to treatment failure was also higher in patients switching to DTG-based dual-drug regimens than INSTI-based 3-drug regimen in a real-world study (13). The persistence of ART in PLWH is influenced by several factors, including side effects and complexity of treatment regimen (14, 15). To understand long-term treatment success, measuring medication persistence, defined as the duration of time from initiation to discontinuation of therapy (16), is important.

The differences in real-world pattern of use and medication persistence between the 2-drug STR and the 3-drug STR have not been well-investigated. This is the first real-world study to directly compare the prescription trends, patients’ characteristics, and medication persistence between DTG/3TC and bictegravir/emtricitabine/TAF (BIC/FTC/TAF), Japan’s most commonly used INSTI-based 3-drug regimen (17) using a large-scale medical claims database in Asia.

## 2 Materials and methods

### 2.1 Study design and data source

This was a retrospective, observational, database study. Study participants were identified from the Medical Data Vision (MDV) database, which is a hospital-based medical claims database in Japan (Medical Data Vision Co., Ltd. Tokyo, Japan). As of April 1, 2023, the MDV database covers 44 million patients from over 390 hospitals in Japan. The database consists of anonymized, individual-level, medical information including patient demographics (age, sex), diagnoses, medical procedures, prescription and hospitalization records, and claims data from all inpatient and outpatient departments.

### 2.2 Study population

#### 2.2.1 Study population [1]

The data of PLWH with a prescription of DTG/3TC or BIC/FTC/TAF since the release date of each STR in Japan were analyzed. The identification period was between January 1, 2018 and December 31, 2021. Patients with records fulfilling all of the following criteria were included: (1) diagnosed with HIV-1 infection according to the International Statistical Classification of Diseases and Related Health Problems, 10th Revision (ICD-10) codes B20-24; (2) had at least two prescription claims for an ART regimen during the identification period; (3) prescribed DTG/3TC or BIC/FTC/TAF. Patients prescribed both DTG/3TC and BIC/TFTC/TAF were excluded.

#### 2.2.2 Study population [2]

In addition to the criteria of the study population [1], the study population [2] was identified based on the following criteria; enrolled in the MDV database for at least 3 months before the index date (the date of the first STR claim within the identification period), and followed-up for at least 6 months after the index date (Supplementary Figure S1). Patients who were prescribed both DTG/3TC and BIC/FTC/TAF were excluded.

### 2.3 Outcomes

The primary outcomes were the differences in clinical characteristics of those who were prescribed DTG/3TC and those who were prescribed BIC/FTC/TAF as the first STR since the release date of each STR, and medication persistence with the DTG/3TC or BIC/FTC/TAF regimen in the study population [2]. The secondary outcomes were as follows: distribution of DTG/3TC, BIC/FTC/TAF, and other ART regimens, which were defined as the final prescribed ART regimen or each patient by year in study population [1] meeting the criteria (1) and (2) sown in the 2.2.1 section; time-to-first prescription since the release date of each STR in the study population [1]; clinical characteristics associated with discontinuation; hospitalization rates in those prescribed DTG/3TC and those prescribed BIC/FTC/TAF; and reasons for the hospitalization in the study population [2].

### 2.4 Definitions

#### 2.4.1 Cohorts

The study population [2] was divided into the following two cohorts; and the (1) DTG/3TC group, defined as taking dolutegravir/lamivudine since its release date, January 31, 2020, in Japan; and the (2) BIC/FTC/TAF group, defined as taking bictegravir/emtricitabine/tenofovir alafenamide since its release date, April 8, 2019, in Japan.

#### 2.4.2 Outcomes

The time-to-first prescription of DTG/3TC or BIC/FTC/TAF was defined as the period from the release date of DTG/3TC or BIC/FTC/TAF in Japan to the date of the first prescription of either DTG/3TC or BIC/FTC/TAF. Medication persistence, defined as the prescribed duration of time from initiation to discontinuation of the index regimen (DTG/3TC or BIC/FTC/TAF), was measured by the number of days from the index date until the end of the supply of the last claim for DTG/3TC or BIC/FTC/TAF, or until the beginning of the first 90-day gap between prescription fills for DTG/3TC or BIC/FTC/TAF, whichever occurred first (Supplementary Figure S1). Hospitalization was defined as any hospitalization that occurred after the first prescription of either DTG/3TC or BIC/FTC/TAF within six months. In any hospitalization, AIDS-related hospitalization was defined as hospitalization due to AIDS-defining cancers/illness defined in the following 2.4.3. section.

#### 2.4.3 AIDS-defining cancers/illness

AIDS-defining cancers/illness were defined as any of the following diseases with ICD-10 codes after the index date: Burkitt lymphoma, non-Hodgkin lymphoma, malignant neoplasm of the cervix uteri, HIV non-tuberculous mycobacteria, HIV cytomegalovirus infection, HIV herpes virus infection, HIV candidiasis, HIV *Pneumocystis carinii* pneumonia, HIV encephalopathy, HIV-associated dementia, HIV lymphoid interstitial pneumonitis, Slim disease, HIV-associated nephropathy, HIV retinopathy, AIDS/acquired immune deficiency syndrome, neonatal HIV infection, AIDS-related complex, acquired immune deficiency syndrome.

#### 2.4.4 Comorbidities

Comorbidities were defined as any of the following disease with ICD-10 codes from the index date: HIV-related disease, hypertension, dyslipidemia, hepatitis B or C coinfection, diabetes mellitus, bone disorder, vascular disease, psychiatric disorders, renal disorders, and malignancies.

The corresponding ICD-10 codes for AIDS-defining cancers/illness and comorbidities are shown in Supplementary Table S1.

### 2.5 Statistical analysis

The proportions of DTG/3TC, BIC/FTC/TAF, and other ART regimens during 2018–2021 were obtained by year. The demographic and clinical characteristics of PLWH were compared between the two cohorts using the *t*-test or the Wilcoxon rank sum test for continuous variables, and Fisher’s exact test for categorical variables. After conducting this analysis, the Mantel–Haenszel test was used to assess the differences in clinical characteristics between the two cohorts by controlling confounding variables. The median time-to-first prescription of DTG/3TC or BIC/FTC/TAF was estimated using Kaplan–Meier curves. The median duration of the regimen was compared between the two cohorts by evaluating continuation rates using Kaplan–Meier methods, with the log-rank test to assess the difference between Kaplan–Meier curves. To estimate the 95% confidence interval (CI), the Greenwood method was used for time-to-first prescription of DTG/3TC or BIC/FTC/TAF and the continuation rate. Taking into account the release date of DTG/3TC in Japan and the end of the identification period, the maximum duration of the index regimen that could be followed-up was 700 days. To identify risk factors associated with discontinuation, univariate Cox proportional hazards models were performed for each cohort separately. Subsequently, variables with a significant association in the univariate analysis were included in multivariable Cox proportional hazards models, adjusting for potential confounders. The difference in hospitalization rates after the first prescription of DTG/3TC or BIC/FTC/TAF was examined. All statistical analyses were performed using R version 4.2.2 software. The remaining statistical tests were two-sided, with significance defined as *p* < 0.005.

This study was conducted in compliance with the Ethical Guidelines for Epidemiological Studies established by the Japanese Government (18) and in accordance with the Declaration of Helsinki of 1975 (revised in 2000) (19).

## 3 Results

### 3.1 Patient selection

A total of 5,088 patients in the MDV database had records of at least two ART prescriptions during the identification period. Of those, 1,529 patients were diagnosed with an AIDS-defining illness during 2008–2021 and prescribed the DTG/3TC or BIC/FTC/TAF regimen. After excluding patients prescribed both STRs, a total of 1,505 patients were identified as the study population [1] including 247 patients prescribed DTG/3TC and 1,258 patients prescribed BC/FTC/TAF (Supplementary Figure S2).

**Figure 1:**
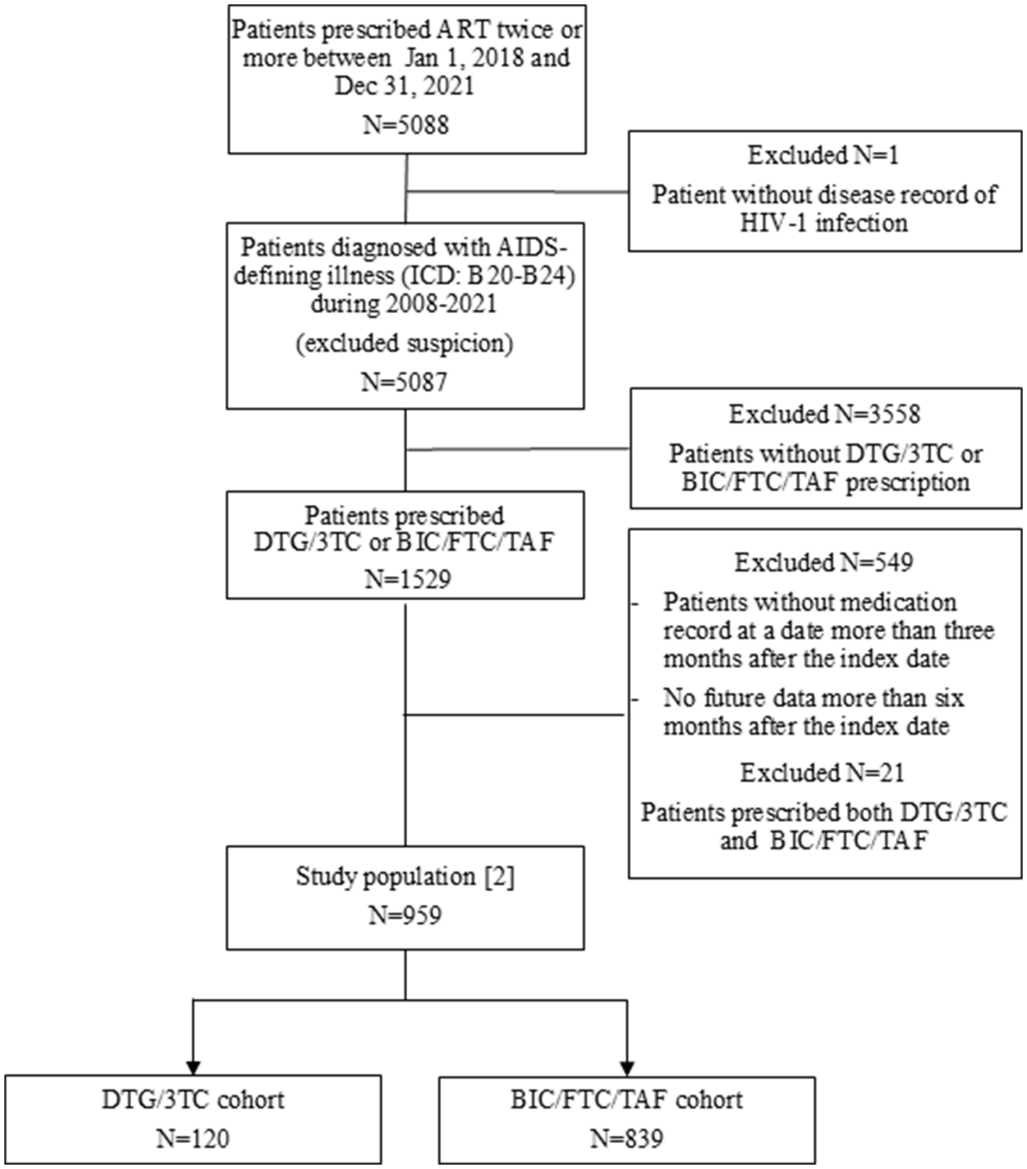
Flowchart for the patient selection method for study population [2] used in this study.

Of 1,529 patients, after excluding patients without medication records 3 months prior to the index date and patients prescribed both STRs, a total of 959 patients were identified as the study population [2]. Eventually, 839 patients and 120 patients were classified into the DTG/3TC and BIC/FTC/TAF cohort, respectively (Figure 1).

### 3.2 Distribution of DTG/3TC, BIC/FTC/TAF, and other ART regimens by year

**Figure 2:**
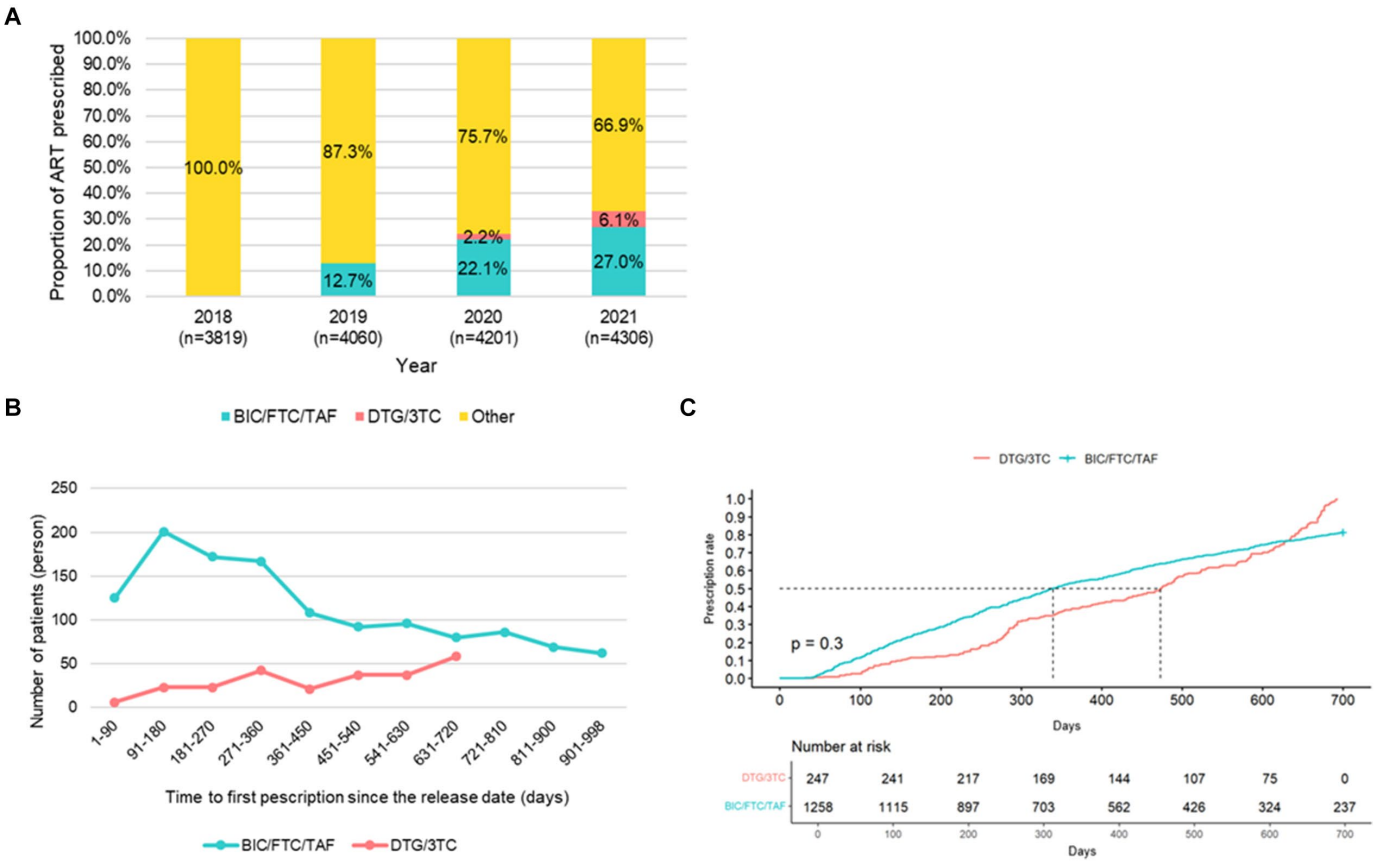
**(A)** Distribution of DTG/3TC, BIC/FTC/TAF, and other ART regimens by year. **(B)** Time-to-first prescription of DTG/3TC and BIC/FTC/TAF since the release date. **(C)** Kaplan-Meir curves of time-to-first prescription of DTG/3TC and BIC/FTC/TAF since the release date.

Of the 5,087 patients diagnosed with an AIDS-defining illness during 2008–2021, the proportion of prescriptions of BIC/FTC/TAF increased steadily from 12.7 to 27.0% since 2019, and that of DTG/3TC increased slightly from 2.2 to 6.1% since 2020 (Figure 2A). On the other hand, the proportion of other ART regimens, which included forty types of drugs in total during 2018–2021, decreased since 2019.

### 3.3 Time-to-first prescription of DTG/3TC and BIC/FTC/TAF since the release date

A total of 1,258 patients and 247 patients were prescribed BIC/FTC/TAF and DTG/3TC, respectively, during the identification period. Overall, 665 patients (52.9%; 665/1258) started BIC/FTC/TAF within one year after the release date of BIC/FTC/TAF. Counter to this trend, patients who started DTG/3TC increased gradually since the release date of DTG/3TC: from six patients within 90 days to 58 patients in 630–720 days (Figure 2B). There was no significant difference in time-to-first prescription since the release date between DTG/3TC and BIC/FTC/TAF (*p* = 0.3) (Figure 2C).

### 3.4 Patients’ characteristics of the two cohorts

Basic characteristics of PLWH patients and differences between the two cohorts are shown in Table 1. There were significant differences in median age, hypertension, dyslipidemia, and diabetes mellitus between the two cohorts. Median follow-up periods were 376 (281–455) days in the DTG/3TC cohort and 693 (505–700) days in the BIC/FTC/TAF cohort, and these were significantly different between the two cohorts (*p* < 0.001). Since mean age was considered a confounding variable, mean age was adjusted in the comparison in the basic characteristics of hypertension, dyslipidemia, diabetes mellitus, and myocardial infraction using the Mantel–Haenszel test. After adjustment for age, the proportion of dyslipidemia was significantly higher in the DTG/3TC cohort than in the BIC/FTC/TAF cohort (*p* = 0.002), but there were no significant differences in hypertension (*p* = 0.51), diabetes mellitus (*p* = 0.057) and myocardial infraction (*p* = 0.14). There were no significant differences in bone disorders, kidney disease, and hepatitis B co-infection (Table 1) and in baseline characteristics of AIDS-related disease carriers between the two cohorts (Table 2).

**Table 1:** Baseline characteristics of PLWH patients.

Characteristic	Overall, *N* = 959	DTG/3TC, *N* = 120	BIC/FTC/TAF, *N* = 839	*p*-value^1^
Age, years (IQR)	44 (36,52)	49 (41,53)	44 (35,52)±12.0	<0.001^2^
**Age group, *n* (%)**				
10–19	2 (0.2%)	0 (0.0%)	2 (0.2%)	
20–29	97 (10.1%)	4 (3.3%)	93 (11.1%)	
30–39	245 (25.5%)	25 (20.8%)	220 (26.2%)	
40–49	318 (33.2%)	37 (30.8%)	281 (33.5%)	
50–59	191 (19.9%)	30 (25.0%)	161 (19.2%)	
60–69	77 (8.0%)	16 (13.3%)	61 (7.3%)	
≥70	29 (3.0%)	8 (6.7%)	21 (2.5%)	
Sex, *n* (%)				0.405
Male	903 (94.2%)	111 (92.5%)	792 (94.4%)	
Female	56 (5.8%)	9 (7.5%)	47 (5.6%)	
Follow-up period, days, median (IQR)	638 (431,700)	367 (281,455)	693 (505,700)	<0.001^2^
Hospitalization (yes), *n* (%)	37 (3.9%)	6 (5.0%)	31 (3.7%)	0.449
Hypertension (yes), *n* (%)	159 (16.6%)	29 (24.2%)	130 (15.5%)	0.025
Dyslipidemia (yes), *n* (%)	240 (25.0%)	50 (41.7%)	190 (22.6%)	<0.001
Diabetes mellitus (yes), *n* (%)	207 (21.6%)	40 (33.3%)	167 (19.9%)	0.001
Bone disorder (yes), *n* (%)	68 (7.1%)	11 (9.2%)	57 (6.8%)	0.342
Vascular disease (yes), *n* (%)	59 (6.2%)	11 (9.2%)	48 (5.7%)	0.154
Myocardial infraction (yes), *n* (%)	3 (0.3%)	2 (1.7%)	1 (0.1%)	0.043
Stroke (yes), *n* (%)	33 (3.4%)	7 (5.8%)	26 (3.1%)	0.173
Angina (yes), *n* (%)	30 (3.1%)	3 (2.5%)	27 (3.2%)	>0.999
Hypertensive heart diseases (yes), *n* (%)	1 (0.1%)	0 (0.0%)	1 (0.1%)	>0.999
Psychiatric disorder (yes), *n* (%)	147 (15.3%)	20 (16.7%)	127 (15.1%)	0.684
Mania and Depression (yes), *n* (%)	92 (9.6%)	132 (10.0%)	80 (9.5%)	0.868
Anxiety (yes), *n* (%)	65 (6.8%)	6 (5.0%)	59 (7.0%)	0.559
Psychosis (yes), *n* (%)	36 (3.8%)	6 (5.0%)	30 (3.6%)	0.439
Dementia (yes), *n* (%)	2 (0.2%)	1 (0.8%)	1 (0.1%)	0.235
Insomnia (yes), *n* (%)	24 (2.5%)	1 (0.8%)	23 (2.7%)	0.347
Kidney disease (yes), *n* (%)	65 (6.8%)	10 (8.3%)	55 (6.6%)	0.44
Chronic renal failure (yes), *n* (%)	41 (4.3%)	7 (5.8%)	34 (4.1%)	0.338
Hemodialysis (yes), *n* (%)	0 (0.0%) 0.0%	0 (0.0%)	0 (0.0%)	
Urolithiasis (yes), *n* (%)	27 (2.8%)	3 (2.5%)	24 (2.7%)	>0.999
Hepatitis B coinfection (yes), *n* (%)	155 (16.2%)	18 (15.0%)	137 (16.3%)	0.792
Hepatitis C co-infection (yes), *n* (%)	85 (8.9%)	5 (4.2%)	80 (9.5%)	0.058
Syphilis, *n* (%)	539 (56.2%)	65 (54.2%)	474 (55.5%)	0.694
Malignancy (yes), *n* (%)	70 (7.3%)	9 (7.5%)	61 (7.3%)	>0.999
AIDS-defining cancers (yes), *n* (%)	48 (5.0%)	4 (3.3%)	44 (5.2%)	0.503
Kaposi sarcoma (yes), *n* (%)	15 (1.6%)	0 (0.0%)	15 (1.8%)	0.239
Burkitt lymphoma (yes), *n* (%)	4 (0.4%)	1 (0.8%)	3 (0.4%)	0.415
Non-Hodgkin lymphoma (yes), *n* (%)	32 (3.3%)	4 (3.3%)	28 (3.3%)	>0.999
Malignant neoplasm of the cervix uteri (yes), *n* (%)	2 (0.2%)	0 (0.0%)	2 (0.2%)	>0.999
Non-AIDS-defining cancers (yes), *n* (%)	29 (3.0%)	5 (4.2%)	24 (2.9%)	0.395

^1Welch’s two sample t-test; Fisher’s exact test.^

^2Wilcoxon rank sum test.^

**Table 2:** Baseline characteristics of AIDS-related disease carriers.

Characteristic	Overall, *N* = 959	DTG/3TC, *N* = 120	BIC/FTC/TAF, *N* = 120	*p*-value^1^
AIDS-defining illnesses, *n* (%)	386 (40.0%)	55 (46.0%)	331 (39.0%)	0.196
HIV non-tuberculous mycobacteria	54 (5.6%)	7 (5.8%)	47 (5.6%)	0.834
HIV cytomegalovirus infection	85 (8.9%)	9 (7.5%)	76 (9.1%)	0.731
HIV herpes virus infection	1 (0.1%)	0 (0.0%)	1 (0.1%)	>0.999
HIV candidiasis	61 (6.4%)	7 (5.8%)	54 (6.4%)	>0.999
HIV *Pneumocystis carinii* pneumonia	191 (20.0%)	27 (23.0%)	164 (20.0%)	0.464
Encephalopathy, *n* (%)	13 (1.4%)	1 (0.8%)	12 (1.4%)	>0.999
HIV encephalopathy	13 (1.3%)	1 (0.8%)	12 (1.4%)	>0.999
HIV-associated dementia	0 (0.0%)	0 (0.0%)	0 (0.0%)	
HIV lymphoid interstitial pneumonitis, *n* (%)	0 (0.0%)	0 (0.0%)	0 (0.0%)	
Slim disease, *n* (%)	0 (0.0%)	0 (0.0%)	0 (0.0%)	
HIV other specified conditions, *n* (%)	50 (5.2%)	3 (2.5%)	47 (5.6%)	0.19
HIV-associated nephropathy	37 (3.9%)	3 (2.5%)	34 (4.1%)	0.611
HIV retinopathy	14 (1.5%)	0 (0.0%)	14 (1.7%)	0.238
HIV disease, *n* (%)	206 (21.0%)	34 (28.0%)	172 (21.0%)	0.057
AIDS Acquired immune deficiency syndrome	205 (21.0%)	34 (28.0%)	171 (20.0%)	0.056
Neonatal HIV infection	0 (0.0%)	0 (0.0%)	0 (0.0%)	
AIDS-related complex	3 (0.3%)	0 (0.0%)	3 (0.4%)	>0.999
AIDS-defining cancers, *n* (%)	48 (5.0%)	4 (3.3%)	44 (5.2%)	0.503

^1Welch’s two-sample t-test; Fisher’s exact test.^

### 3.5 Medication persistence of the two cohorts

**Figure 3:**
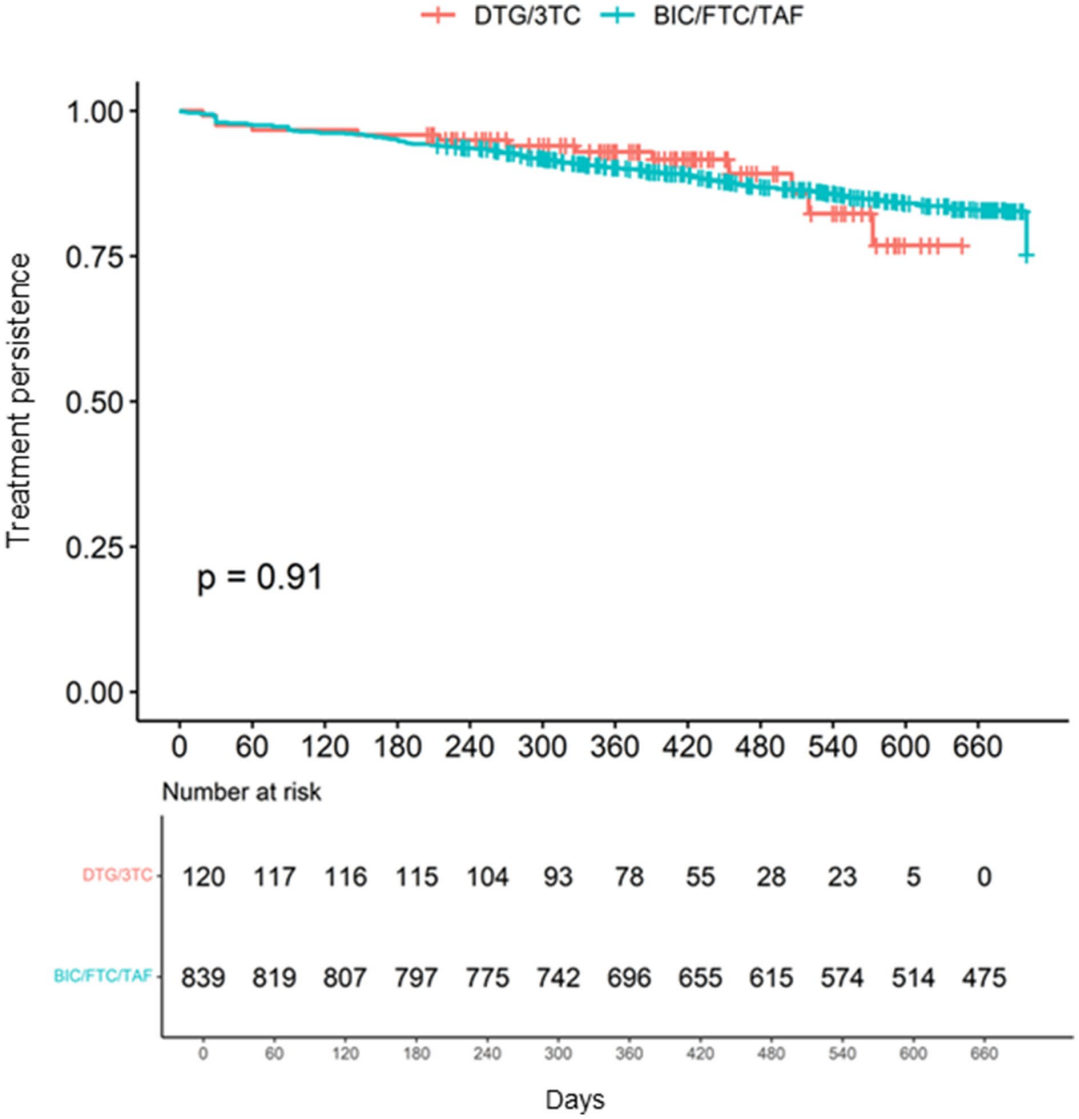
Medication persistence of the DTG/3TC and BIC/FTC/TAF cohorts up to 700 days.

There was no significant difference in medication persistence up to 700 days between the two cohorts (log-rank test *p* = 0.91) (Figure 3). Hazard ratios of patients’ characteristics for discontinuation in each cohort are shown in Supplementary Figure S3.

### 3.6 Hospitalization

Hospitalizations were recorded for six patients (5.0%; 6/120) in the DTG/3TC cohort and 31 patients (3.7%; 31/839) in the BIC/FTC/TAF cohort within 6 months of the first prescription of DTG/3TC or BIC/FTC/TAF. AIDS-related hospitalization occurred in one patient (16.7%; 1/6) in the DTG/3TC cohort and 12 patients (38.7%; 12/31) in the BIC/FTC/TAF cohort. The reasons for AIDS-related hospitalization in each cohort were as follows: HIV in one patient of the DTG/3TC cohort, and HIV in nine patients and Burkitt lymphoma in three patients of the BIC/FTC/TAF cohort. There were no deaths in either cohort.

## 4 Discussion

We conducted this retrospective, cohort study to explore appropriate ART regimen for PLWH in Asian population where multiple morbidities would increase due to aging. This is the first study which provides real-world data directly comparing the trend of DTG/3TC prescription and its medication persistence with that of BIC/FTC/TAF using a Japanese medical claim database. The results showed that the proportion of prescriptions with DTG/3TC increased recently, as rapidly as did that of BIC/FTC/TAF since the release date, instead of other ART regimens. Our previous studies showed a higher prevalence of comorbidities in PLWH than in people without HIV with the increase of age, which likely strongly affected selection of the ART regimen (4, 5). The upward trend in the prescription of DTG/3TC may suggest that the DTG/3TC is selected for elderly PLWH with multiple morbidities to reduce long-term adverse effects and potential drug–drug interactions due to polypharmacy, as well as to improve adherence, which aligns with guideline recommendations (20–22). With the increase in the numbers of PLWH and multiple morbidities due to further aging of society, it is expected that HIV medication provided by not only HIV infectious disease specialists, but also general or primary care physicians could increase in Japan. Since the present study demonstrated that DTG/3TC was prioritized for elderly PLWH and those with chronic comorbidities, this large-scale database study provides information for general or primary care physicians to treat PLWH with a simplified ART regimen. In addition, healthcare costs related to DTG/3TC were lower than those of other 3- or 4-drug regimens. DTG/3TC could be beneficial in a super-aging society such as Japan, with an upsurge in healthcare costs (23, 24). Some studies have investigated the effectiveness and safety of DTG/3TC in real-world clinical practice in Asian countries (25, 26), but sample size was limited. The present study is the first large-scale database study in Japan.

In the GEMINI trial involving treatment-naïve patients, DTG/3TC did not demonstrate improvements in lipid profiles (8), but the TANGO trial involving treatment-experienced patients demonstrated better changes from baseline in the lipid values with DTG/3TC compared to TAF-based regimens at 144 weeks (11). Switching from TAF-based regimens may improve lipid metabolism. In fact, the present study showed that prescription of DTG/3TC was associated with PLWH patients with higher age and dyslipidemia. Similar results were reported from the large cohort study in the USA (27) A retrospective study from China showed that DTG/3TC was selected over BC/FTC/TAF for elderly patients and those with hypertension and diabetes mellitus (28). In the present study, although there were no significant differences in hypertension, diabetes mellitus, myocardial infraction after adjusting age, and bone disorders and kidney disease between the two cohorts, the prevalence of each was numerically higher in the DTG/3TC cohort than in the BIC/FTC/TAF cohort. In GEMINI trial, adverse events related to the kidneys and bones were less frequent in the DTG/3TC group (8). DTG has low potential for drug interactions because DTG is primarily metabolized by uridine diphosphate glucuronosyltransferase 1A1 (UGT1A1) and, to a lesser extent, by cytochrome P450 (CYP)-3A4 (29). These backgrounds may contribute to drug selection. However, further investigation of medication preference is needed in real-world practice. The guidelines recommend a TAF-based regimen for patients with hepatitis B co-infection (19–21), and they suggest that DTG/3TC should be used for ART-naive patients who are HBsAg-negative. Previous cohort studies from other countries showed that treatment-naïve or treatment-experienced PLWH and with hepatitis B co-infection accounted for 0% in the DTG/3TC cohort (24, 30), whereas a cohort study from Japan showed that, of treatment-experienced PLWH with DTG/3TC switched from 3-drug regimens, more than half of the patients had hepatitis B co-infection (31). In the present study, there was no significant difference in the prevalence of hepatitis B co-infection between the two cohorts, suggesting that patients’ background characteristics with multiple morbidities and polypharmacy may affect physicians’ choices more rather than hepatitis B co-infection.

Medication persistence was not significantly different between the DTG/3TC and BIC/FTC/TAF cohorts in the present study. Additionally, the rates of hospitalization and AIDS-related hospitalization were similar between the two cohorts. However, due to the limited number of hospitalization events, further investigation is needed to assess properly the matter. The indirect comparison of clinical trials between DTG/3TC and 3-drug single-tablet regimens (BIC/FTC/TAF and DTG/ABC/3TC) in therapy-naïve PLWH at 144 weeks showed that frequencies of discontinuation and adverse events were comparable across all regimens (32). Real-world studies from Europe showed that the durability of treatment was similar in the DTG/3TC and BIC/FTC/TAF groups (30, 33). In our study, the difference in the median follow-up period between the two cohorts may affect the accurate evaluation of persistence, thus, longer-term observation needs to be assessed. The retrospective study from China showed that the virological and immunological efficacy were similar in DTG/3TC and BIC/FTC/TAF for 48 weeks, and both regimens were well-tolerated and safe (28). The post-marketing surveillance of DTG/3TC conducted in Japan reported no new concerns about safety and effectiveness (34). These results indicate that DTG/3TC may have a high barrier to resistance even though it is a 2-drug regimen as well as maintaining safety. Previous studies from Europe and South Africa reported that cross-resistance occurred in sequential use of INSTIs due to resistance-associated mutations, however, the susceptibility to DTG was higher than other INSTIs (35–37). In addition to effectiveness and safety, medication persistence in PLWH is influenced by other factors, such as psychological and economic factors (14, 15), however, our study did not investigate them.

This study has several limitations. First, the database lacks laboratory data, such as CD4+ cell counts, which are related to the parameters of viral suppression, viral load, genotypic resistance test, and HBV serology status. Although numerical information related to virological failure could not be obtained, medication persistence and hospitalization rates were compared in the two cohorts to investigate tolerability. However, since clinical characteristics associated with the treatment discontinuation and hospitalization were not clarified, it is difficult to directly link these to virological and immunological effectiveness. Second, since the database was originally recorded for billing purposes, the reasons for discontinuations, such as virological failure or adverse events, were not clarified. Third, since the database is hospital-based, the medical history could not be completely captured. For instance, patient transfers made it impossible to link the database with the medical records. Thus, the interpretation of the results in the present study might be limited to clinical practice in Japan. Although this study has these limitations, it included a high number of subjects, which allowed the comparison of the real-world treatment patterns and medication persistence between the two cohorts.

## 5 Conclusion

We conducted this retrospective, cohort study among Asian PLWH. DTG/3TC was more likely to be selected than BIC/FTC/TAF in clinical practice for elderly patients and those with chronic disease, in accordance with the treatment strategy recommended by guidelines. Comparable medication persistence observed for the DTG/3TC and BIC/FTC/TAF regimens may be attributed to appropriate drug selection for PLWH patients to alleviate concerns of drug–drug interactions, and long-term toxicity. The 2-drug single-tablet regimen DTG/3TC has become an important ART regimen for PLWH with age-associated multiple morbidities and polypharmacy in the aging society.

## Data Availability

The raw data supporting the conclusions of this article will be made available by the authors, without undue reservation.
