# Corrigendum: Characteristics and real-world medication persistence of people living with HIV treated with DTG/3TC or BIC/FTC/TAF: a hospital claims database study in Japan

**DOI:** 10.3389/fmed.2025.1555353

**Published:** 2025-02-10

**Authors:** Rie Kanamori, Nozomi Aoki, Akio Kanazawa, Mayumi Yuda, Nao Makino, Emi Ohata, Nobuyuki Fukui, Hirotake Mori, Hirohide Yokokawa, Toshio Naito

**Affiliations:** ^1^Department of General Medicine, Juntendo University Faculty of Medicine, Tokyo, Japan; ^2^Center for Promotion of Data Science, Juntendo University Graduate School of Medicine, Tokyo, Japan

**Keywords:** HIV, antiretroviral therapy, single-tablet regimen, treatment pattern, medication persistence, real-world, characteristics, aging society

In the published article, there was an error in the author list, and author Daisuke Nakamoto was erroneously included. The corrected author list and Author Contributions appear below.

Rie Kanamori, Nozomi Aoki, Akio Kanazawa, Mayumi Yuda, Nao Makino, Emi Ohata, Nobuyuki Fukui, Hirotake Mori, Hirohide Yokokawa, Toshio Naito

