# Supplementary figures and images for: Characteristics and real-world medication persistence of people living with HIV treated with DTG/3TC or BIC/FTC/TAF: a hospital claims database study in Japan
Source: Front Med (Lausanne). 2024 Sep 10;11:1329922. doi: 10.3389/fmed.2024.1329922 (PMC11420020; doi:10.3389/fmed.2024.1329922)

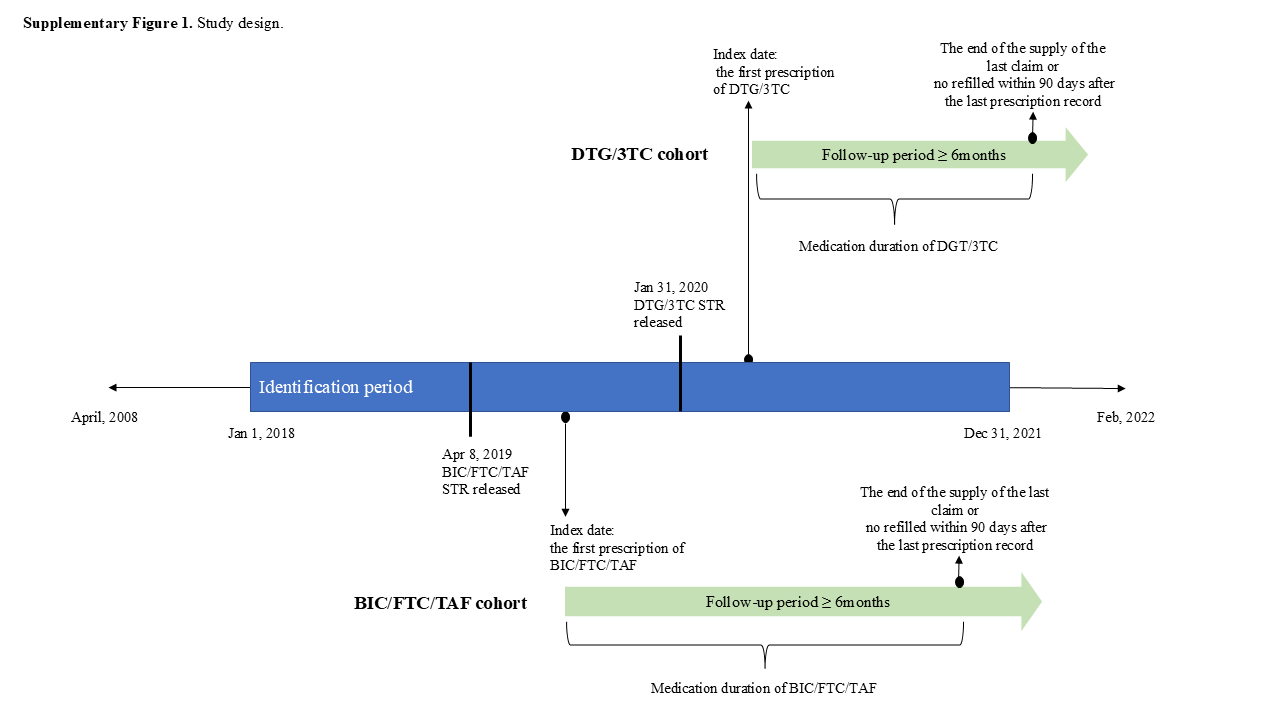

Supplement: Supplementary file 1 [file Image_1.TIF]

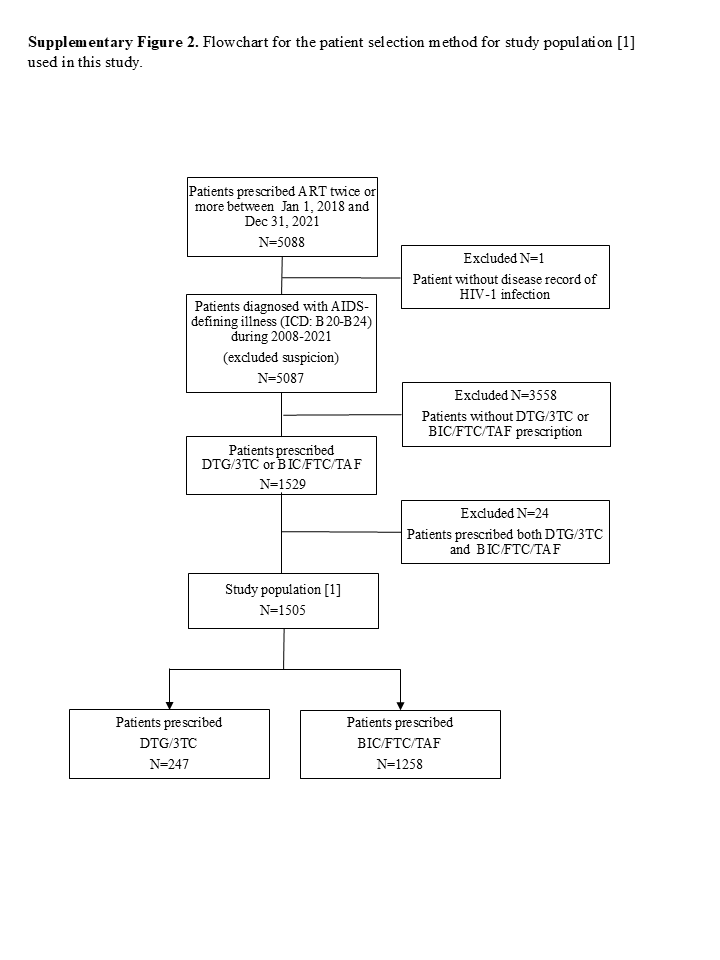

Supplement: Supplementary file 2 [file Image_2.TIF]

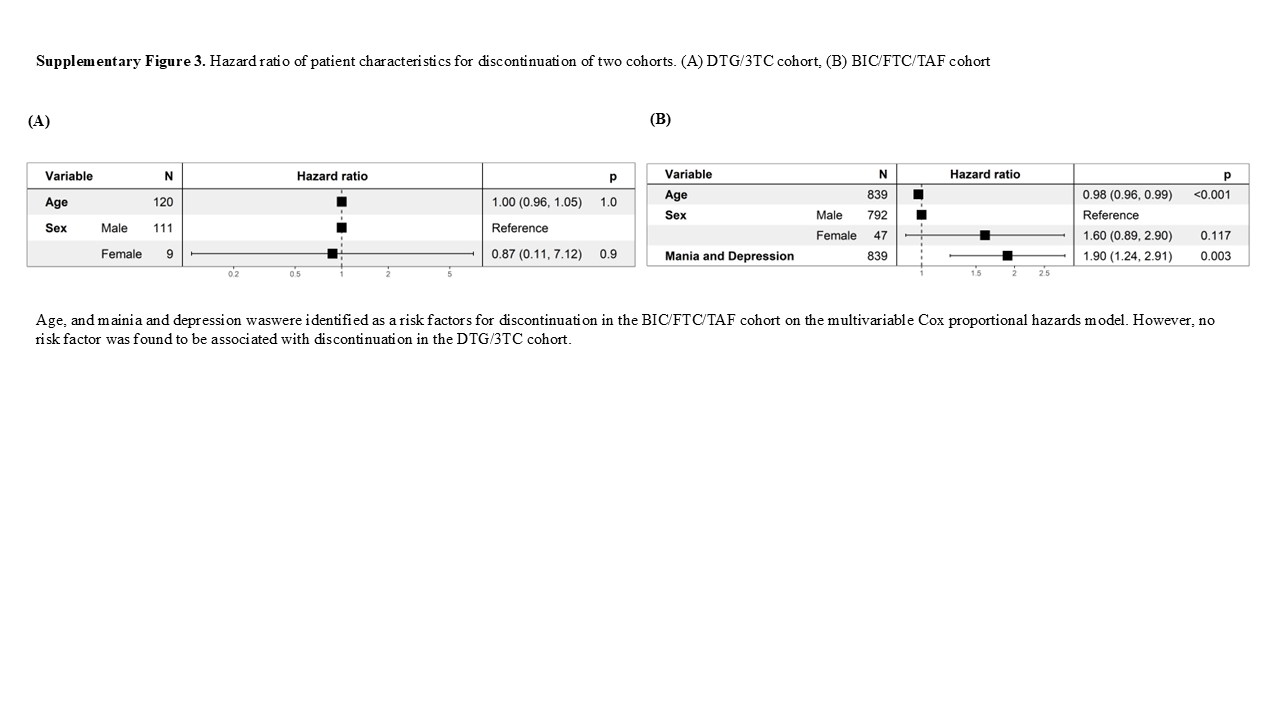

Supplement: Supplementary file 3 [file Image_3.TIF]
