# Supplementary material for: Characteristics and real-world medication persistence of people living with HIV treated with DTG/3TC or BIC/FTC/TAF: a hospital claims database study in Japan
Source: Front Med (Lausanne). 2024 Sep 10;11:1329922. doi: 10.3389/fmed.2024.1329922 (PMC11420020; doi:10.3389/fmed.2024.1329922)
Supplement: Supplementary file 4 [file Table_1.DOCX]

**Supplementary Table 1. ICD-10 codes to identify AIDS-defining illness and comorbidities.**

| Disease name | ICD-10 code |
| --- | --- |
| Type 2 diabetes | E11 - E14 |
| Hypercholesterolaemia or hyperlipidaemia | E78.0 - E78.5 |
| Hypertension | I10、I12、I14 - I15 |
| Hepatitis B infection | B18.1 |
| Hepatitis C infection | B18.2 |
| Mania and depression | F30 - F32 |
| Psychiatric disorders |  |
| Psychosis | F2 |
| Anxiety | F40 - F41 |
| Insomnia | F51 |
| Dementia | F01、F03 |
| Bone disorder | M80 - M81 |
| Angina | I20 |
| Vascular disease |  |
| Myocardial infarction | I21 - I22 |
| Stroke | Designated by codes for health insurance claim |
| Hypertensive heart diseases | I11 |
| Kidney disease | N18 - N19 |
| Chronic kidney disease |  |
| Hemodialysis | Designated by codes for health insurance claim |
| Urolithiasis | N20 - N21 |
| Syphilis | A51 - A53 |
| Malignancies | B21.0 - B21.2 C00 - C97 |
| AIDS-defining cancers | B21.0, C46 |
| Burkitt lymphoma | B21.1, C83.7 |
| Non-Hodgkin lymphoma | B21.2, C82 - C85, excludingC83.7 |
| Malignant neoplasm of the cervix uteri | C53 |
| Non-AIDS-defining cancers | C00 - C97, excluding codes for AIDS-defining cancer |
| AIDS-defining illnesses |  |
| HIV non-tuberculous mycobacteria | B200, A31 |
| HIV cytomegalovirus infection | B202, B25 |
| HIV herpes vires infection | B203 |
| HIV candidiasis | B204, B378 |
| HIV Pneumocystis carinii pneumonia | B20, B59 |
| Kaposi sarcoma | B21.0, C46 |
| Burkitt lymphoma | B21.1, C83.7 |
| Non-Hodgkin lymphoma | B21.2, C82 - C85, excluding C83.7 |
| HIV encephalopathy | B220-8830098 |
| HIV-associated dementia | B220-8845516 |
| HIV lymphoid interstitial pneumonitis | B221 |
| Slim disease | B222 |
| HIV-associated Nephropathy | B238-8830097 |
| HIV retinopathy | B238-8844004 |
| AIDS | B24-2793011 |
| Neonatal HIV infection | B24-7712015 |
| AIDS-related complex | B24-8830055 |
| Acquired immune deficiency syndrome | B24-2793007 |
| Malignant neoplasm of the cervix uteri | C53 |
